# Nerve Growth Factor-Based Therapy in Alzheimer’s Disease and Age-Related Macular Degeneration

**DOI:** 10.3389/fnins.2021.735928

**Published:** 2021-09-09

**Authors:** Giuseppina Amadoro, Valentina Latina, Bijorn Omar Balzamino, Rosanna Squitti, Monica Varano, Pietro Calissano, Alessandra Micera

**Affiliations:** ^1^Institute of Translational Pharmacology (IFT)-CNR, Rome, Italy; ^2^European Brain Research Institute, Rome, Italy; ^3^Research Laboratories in Ophthalmology, IRCCS-Fondazione Bietti, Rome, Italy; ^4^Molecular Markers Laboratory, IRCCS Istituto Centro San Giovanni di Dio Fatebenefratelli, Brescia, Italy

**Keywords:** neuroprotection, brain degeneration, trace metals, Alzheimer’s disease, age-related macular degeneration, nerve growth factor, retinal degeneration, biomarkers

## Abstract

Alzheimer’s disease (AD) is an age-associated neurodegenerative disease which is the most common cause of dementia among the elderly. Imbalance in nerve growth factor (NGF) signaling, metabolism, and/or defect in NGF transport to the basal forebrain cholinergic neurons occurs in patients affected with AD. According to the cholinergic hypothesis, an early and progressive synaptic and neuronal loss in a vulnerable population of basal forebrain involved in memory and learning processes leads to degeneration of cortical and hippocampal projections followed by cognitive impairment with accumulation of misfolded/aggregated Aβ and tau protein. The neuroprotective and regenerative effects of NGF on cholinergic neurons have been largely demonstrated, both in animal models of AD and in living patients. However, the development of this neurotrophin as a disease-modifying therapy in humans is challenged by both delivery limitations (inability to cross the blood–brain barrier (BBB), poor pharmacokinetic profile) and unwanted side effects (pain and weight loss). Age-related macular degeneration (AMD) is a retinal disease which represents the major cause of blindness in developed countries and shares several clinical and pathological features with AD, including alterations in NGF transduction pathways. Interestingly, nerve fiber layer thinning, degeneration of retinal ganglion cells and changes of vascular parameters, aggregation of Aβ and tau protein, and apoptosis also occur in the retina of both AD and AMD. A protective effect of ocular administration of NGF on both photoreceptor and retinal ganglion cell degeneration has been recently described. Besides, the current knowledge about the detection of essential trace metals associated with AD and AMD and their changes depending on the severity of diseases, either systemic or locally detected, further pave the way for a promising diagnostic approach. This review is aimed at describing the employment of NGF as a common therapeutic approach to AMD and AD and the diagnostic power of detection of essential trace metals associated with both diseases. The multiple approaches employed to allow a sustained release/targeting of NGF to the brain and its neurosensorial ocular extensions will be also discussed, highlighting innovative technologies and future translational prospects.

## Introduction

As an integral part/extension of the central nervous system (CNS), ocular structures display several cytological, anatomical, and developmental similarities with the brain so that the retina and cerebral areas, receiving directly (visual system) and indirectly (amygdala, hippocampus, and other hypothalamic nuclei) its inputs, constitute the so-called “eye–brain connection” (Erskine and Herrera, 2014; Tirassa et al., 2018). Because of the biological connection between the brain and eyes, analogous and corresponding pathological processes can lead to dysfunction of both of them. Oxidative injury, chronic activation of inflammatory pathway(s), mitochondrial impairment, vascular changes, neurotrophin(s) imbalance, protein quality control deficits and proteostasis, metal ion dyshomeostasis, genetics causes, or a combination of them are proposed to be involved in age-related disorders featured by brain and retinal degeneration, including Alzheimer’s disease (AD) and age-related macular degeneration (AMD; Kaarniranta et al., 2011; Masuzzo et al., 2016; Xu et al., 2018; Acevedo et al., 2019; Micera et al., 2019; Mufson et al., 2019; Wang and Mao, 2021). AD is a complex and multifactorial neurodegenerative disorder representing the main cause of dementia in the elderly (Walsh and Selkoe, 2004; Querfurth and LaFerla, 2010). The extracellular neuritic plaques, composed of β-amyloid (Aβ), and the intracellular neurofibrillary tangles (NFTs), composed of hyperphosphorylated tau protein, are the two key neuropathological hallmarks associated with this disease. Both Aβ plaques and NFTs impair synaptic plasticity and neural circuit networks causing the clinical symptoms of progressive memory loss (Spires-Jones and Hyman, 2014; Masters et al., 2015). AMD is a late-onset retinal neurodegenerative disorder representing the major cause of blindness in Western countries (Fine et al., 2000; Gehrs et al., 2006; Jager et al., 2008; Çerman et al., 2015). Accumulation of insoluble extracellular aggregates called drusen in the retina, degeneration of retinal pigment epithelium (RPE) cells, and choroidal neovascularization (CNV) manifest in AMD-suffering subjects leading to irreversible loss of vision (Ambati and Fowler, 2012; van Lookeren Campagne et al., 2014).

Here, we discuss how the understanding of the common pathophysiological molecular mechanisms linking these two overlapping disorders will help in the development of novel diagnostic/prognostic biomarkers and effective therapeutic avenues. Furthermore, the power of the nerve growth factor (NGF) in maintaining the functional phenotype(s) of different neuronal populations and retinal cells along with the recent optimization of innovative strategies allowing both local and systemic delivery of this neurotrophin jointly prospects its utilization as a promising, neuroprotective option for the treatment of both AD and AMD.

## Alzheimer’s Disease and AMD Pathophysiology: Similarities and Differences

**FIGURE 1:**
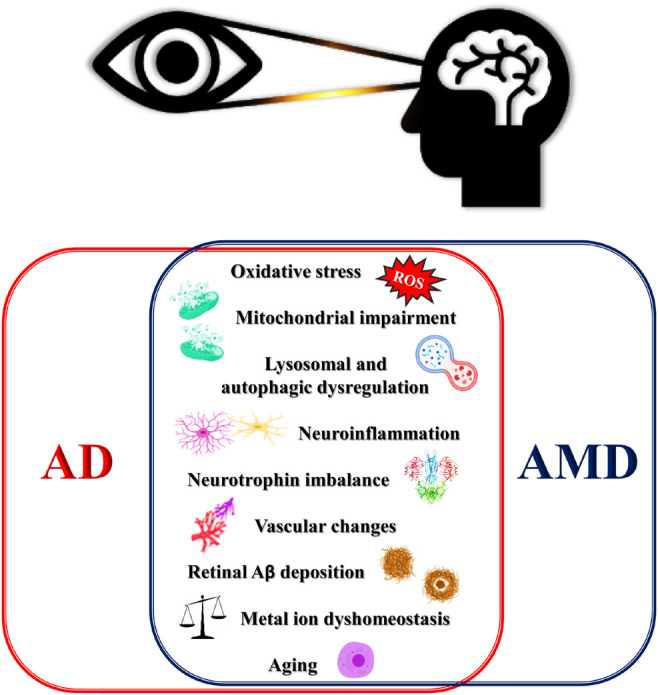
Schematic for overlapping degenerative mechanisms shared by AMD and AD. These pathways have been associated with brain and retina degeneration, cognitive, deficits and loss of vision. AD, Alzheimer’s disease; AMD, age-related macular degeneration; ROS, reactive oxygen species.

Epidemiological, genetic, pathological, and clinical lines of evidence have documented a strong relationship between AD and AMD (Wang and Mao, 2021) which have prompted scientists to speculate that AMD could be a form of “Alzheimer’s disease in the eye” (Kaarniranta et al., 2011; Ohno-Matsui, 2011) (Figure 1).

To better understand the burden on public health of these two age-related multifactorial neurodegenerative disorders, it is worth pointing out that the prevalence of AD is estimated to be 46 million of cases worldwide (Brookmeyer et al., 2007; Reitz and Mayeux, 2014), with the number of AD patients expected to triple in 2050 (Baumgart et al., 2015), reaching 131 million of cases (Prince et al., 2013). In a similar way, 15% (age 65–74), 25% (age 75–84), and 30% (age 85% or more) of subjects are affected with AMD (Klein et al., 2004; Wong et al., 2014). Indeed, investigations have demonstrated that, in addition to a strong genetic component, aging is the principal common risk factor for both AD and AMD (Coleman et al., 2008; Mayeux and Stern, 2012; Rudnicka et al., 2012). Genetic heritable components play a role in the development of AD, with rare autosomal mutations in the three genes *APP*, *PSEN1*, and *PSEN2* and the APOE epsilon-4 allele associated with familial early-onset and sporadic late-onset AD forms, respectively (Mayeux and Stern, 2012). On the other hand, CFH (encoding for a complement inhibitor factor) and ARMS2/HTRA1 (encoding for a serine protease) are linked with AMD (Logue et al., 2014; Woo et al., 2015; Tsiloulis et al., 2016). Interestingly, genome-wide association studies (GWAS) have shown that single nucleotide polymorphisms (SNPs) in *ABCA7* (encoding for ATP-binding cassette transporter subfamily A member 7), *HGS* (encoding for a member of the clathrin-mediated endocytosis signaling pathway), and *PILRA/ZCW1P1* genes (encoding for the paired-immunoglobulin-like type 2 receptor which binds with herpes simplex virus-1 and for a zinc finger protein functioning as a histone modification, respectively) are also common in the pathogenesis of both AMD and AD (Ding et al., 2009; Kaarniranta et al., 2011). Besides, hypercholesterolemia, hypertension, atherosclerosis, obesity, diabetes, and environmental risk factors also strongly contribute to the development of both AD and AMD in mid-life (Kivipelto et al., 2001, 2002, 2005; Newman et al., 2005; Polidori et al., 2001; Seddon et al., 2005; Age-Related Eye Disease Study Research Group (AREDSRG), 2000; Kaarniranta et al., 2011; Ohno-Matsui, 2011). Relevantly, patients suffering AMD exhibit an increased risk of developing AD as compared with people without AMD (Frost et al., 2016; Wen et al., 2021), even though a direct association between these two disorders has not been confirmed (Williams et al., 2014). Besides, compelling studies have demonstrated that vision abnormalities featured by degeneration of the visual cortex and/or RGC loss or AMD-like retinal degeneration are prominent in AD patients and manifest even before clinical signs of cognitive decline (Ashok et al., 2020).

The phenotypic parallelism between AD and AMD is strongly highlighted by experimental evidence that the beta-amyloid (Aβ) peptide – the main biochemical component of extracellular senile plaques featuring the AD brain – is also found in ocular drusen deposits characterizing AMD (Anderson et al., 2004). In particular, these latter inclusions generally localized in the macula, the central region of the retina, are focal depositions of acellular debris accumulating between the basal lamina of postmitotic neuroepithelial cells called RPE – which take care of neural cells, rods, and cones – and the inner collagenous layer of the Bruch’s membrane (Usui et al., 2018; Blasiak, 2020). The majority of patients manifest a dry form of AMD which is characterized by the presence of amorphic drusens classified as “hard” and “soft” drusen. “Hard” drusen is focal and thickening of the basement membrane of RPE, whereas “soft” drusen is hardening confined to the separation of the basement membrane from Bruch’s membrane at the inner collagenous zone. On the other hand, pseudodrusen, which is associated with clinical progression to more severe stages of AMD, is a deposit located above the basement membrane of RPE (Miller, 2013). Advanced AMD manifests with progressive RPE deterioration and develops into the exudative AMD (eAMD) with CNV or non-exudative AMD (neAMD) with geographic atrophy (GA) in which drusen coalesces and causes damage to RPE and photoreceptors (Jindal, 2015; Hernandez-Zimbron et al., 2018). Interestingly, the amyloid deposits possessing a central core with non-radiating fibrils have been also detected in the inner layers of the retina of AD patients, in contrast to classical/neuritic plaques found in their brain (Koronyo-Hamaoui et al., 2011). Relevantly, just as in AD, the role of these deposits is controversial in AMD and other types of macular degeneration since it is still unclear whether they are causing the RPE dysfunction or are a mere consequence of the impairment of the RPE function (Biscetti et al., 2017). In addition to Aβ, several protein and lipid constituents of drusens – including clusterin, vitronectin, amyloid P, esterified cholesterol and phosphatidylcholine, apolipoprotein E, and inflammatory mediators, such as acute phase reactants and complement components (C5, C5b9, and C3 fragments) – are also present in insoluble cerebral aggregates of AD (Mullins et al., 2000; Crabb et al., 2002; Luibl et al., 2006; Isas et al., 2010; Wang et al., 2010). Likewise, metal elements such as Zn^2+^, Fe^3+^, Cu^2+^, ubiquitin, and lipofuscin account for the biochemical composition of lesions from both AD and AMD (Atwood et al., 2002; Ryhänen et al., 2009; Ohno-Matsui, 2011). Although rodents have neither functional macula nor sharp vision in the retina, an important demonstration of commonalities occurring between AMD and AD is represented by the human APOE4-expressing transgenic mice, an animal model carrying the APOE4 allelic variant which is one of the major risk factors for AD onset/progression. Interestingly, old animals of this strain, when fed a high-fat diet, exhibit an ocular phenotype which resembles several crucial features of AMD. Besides, their retinal as well as memory dysfunction is strongly attenuated by the delivery of antibody Aβ-neutralizing (Ding et al., 2008, 2011). Additional parallel pathophysiological events occurring in both AMD and AD pathogenesis encompass increased oxidative stress and mitochondrial and lysosomal dysfunctions. Owing to the relative enrichment of unsaturated lipids, the abundance of redox-active transition metals, the modest antioxidant defense, the neurotransmitter auto-oxidation and RNA oxidation, and the Ca^2+^ signaling along with the high energetic demand, the brain is largely susceptible to oxidative stress especially at terminal synaptic ends (Cobley et al., 2018). In a corresponding manner, the retina is particularly prone to undergo injury linked to excessive generation of reactive oxygen species (ROS) due to its high oxygen consumption, exposure to continuous light, high intracellular levels of polyunsaturated fatty acids (PUFAs) in photoreceptor outer segments, presence of photosensitizers in the RPE and neurosensory retina, and daily phagocytosis of the retinal outer segment originating from rods and cones (Beatty et al., 2000; Fine et al., 2000; Klein et al., 2004; Buch, 2005; Gehrs et al., 2006; Katta et al., 2009). Thus, it is not surprising that proteins isolated from RPE cells are heavily modified by oxidative stress markers, including malondialdehyde, 4-hydroxynonenal, AGE, and RAGE modifications (Schutt et al., 2002, 2003). Degradation-resistant aggregates of lipofuscin consisting of cross-linked, oxidized proteins and lipids also accumulate in the brain and in the eye of AD and AMD in association with the occurrence of pro-oxidant environmental conditions, especially those caused by light stimulation (Kaarniranta et al., 2011). Damage of mtDNA, which is more vulnerable than nuclear DNA to damage from oxidation and blue light, is proven to accumulate during aging in the retina along with changes in morphology and function of respiration-competent mitochondria (Barreau et al., 1996; Ballinger et al., 1999; Barron et al., 2001; Liang and Godley, 2003; King et al., 2004; Godley et al., 2005; Feher et al., 2006). Correspondingly, mitochondrial dysfunction and oxidative injury marked by peroxidation, nitration, reactive carbonyls, and nucleic acid oxidation are early and prominent into selectively damaged hippocampal and cortical neuronal populations in AD brain (Nunomura et al., 2001; Ganguly et al., 2021; Ke et al., 2021). The autophagic, lysosomal, and proteasomal signal transduction pathways, the main protein quality control mechanisms endowed with the capacity of repairing oxidative stress-induced damages, are also altered both in AMD eyes and AD brain (Kaarniranta et al., 2011). This imbalance in clearance systems likely causes an intracellular buildup in misfolded/damaged proteins, including Aβ and phospho-tau (ptau; Loeffler et al., 1993; Johnson et al., 2002; Ohno-Matsui, 2011), which ends up in the formation of detrimental insoluble aggregates in the brain and in the eye (Kaarniranta et al., 2010; Bruni et al., 2020), as largely shared both in these neurodegenerative diseases. Impaired or insufficient autophagy activity is implicated in numerous age-related degenerative diseases, including AMD (Kaarniranta et al., 2011) and AD (Rami, 2009; Wong and Cuervo, 2010), since postmitotic, terminally differentiated neurons are largely sensitive to stress in degradative intracellular pathways leading to proteostasis. The neurotoxic intracellular Aβ, present in late endosomes and lysosome compartments of retinal neurons from aged familial AD mice models showing AMD pathology, is more likely to undergo release into cytosolic compartment by provoking destabilization/leaking of the lysosome membrane (Park et al., 2017; Habiba et al., 2020). This further corroborates the finding that an impaired autophagy is involved in Aβ-dependent eye neurodegeneration (Golestaneh et al., 2017). Markers of activated autophagy colocalize with Aβ deposits, especially in aged human RPE cells and specimens from postmortem AMD subjects, and clearly display impairment of an autophagic pathway in the retina (Mitter et al., 2014). In transgenic animal models expressing E693Δ mutation (referred to as the “Osaka” mutation) of amyloid precursor protein (APP), the age-dependent accumulation of intraneuronal Aβ oligomers causes leakage of cathepsin D from endosomes/lysosomes into the cytoplasm, cytochrome c release from the mitochondria, and activation of caspase-3 in the hippocampi of 18-month-old mice (Umeda et al., 2011) as well. Neuroinflammation is considered to crucially take part in neuronal loss and deposition of extracellular aggregates both in AD retina and brain. In particular, microglial cells, which are phagocytes of the CNS, have functional similarities with RPE cells, macrophages, or dendritic cells in AMD, and when activated, all of them secrete similar inflammatory mediators. The phagocytic activity of reactive microglial cells engulfing and degrading Aβ is progressively lost in close proximity to the fibrillary plaque into the brains of AD patients, while chronically activated neuroglia start to release damaging chemokines and cytokines – notably IL-1, IL-6, and tumor necrosis factor α – which further contribute to affect the protein degradation systems and neuronal viability. Likewise, under pathological conditions, microglia migrating into the subretinal space from the inner retina is early activated by Aβ deposited in the drusen with the production of different types of neurotoxic cytokines which drive both the protein aggregation and cell death during development and progression of AMD (Ohno-Matsui, 2011). Astrocyte activation is also present around drusens and in the subretinal space and senile plaques in AMD and AD specimens (Sivak, 2013). In addition to chronic oxidative stress, complement activation is a central mechanism in both pathologies with classical and alternative pathways preferentially involved in AD and AMD, respectively (McGeer et al., 2005; McGeer and McGeer, 2010). Colocalization of Aβ and iC3b immunoreactivity have been detected in the amyloid vesicles within the drusens with markers of chronic complement activation, such as C3d and C4 being elevated in the plasma of both AMD and AD patients (Ohno-Matsui, 2011). Proteins of the acute phase can be found both in the drusens and senile plaques (McGeer and McGeer, 2004; Donoso et al., 2006). Due to oxidative stress and inflammation, secondary neovascularization specifically manifests in exudative AMD with new vessels sprouting from the choroidal capillaries through the Bruch’s membrane into the sub-RPE space or into the retinal layer (Miller, 2013; Wang and Mao, 2021). Aβ accumulation, alterations of blood flow dynamics, and an increased endothelial cell apoptosis have been detected in the retina and choroidal vessels of complement factor H knockout mice [Cfh(–/–)], suggested as a valuable model for AMD mice (Aboelnour et al., 2016). Similarly, angiogenesis hallmark is featured in the hippocampus of postmortem human brain tissues from patients with AD by an elevated immunoreactivity of integrin aVb3, a dimeric glycoprotein expressed on the endothelial cell surface markedly increased on angiogenic vessels (Boscolo et al., 2007; Desai et al., 2009). Nevertheless, even though many parallel pathophysiological aspects are shared by both AMD and AD, several concerns have challenged the development of common biomarkers and effective therapies for their clinical management. For instance, the genetic background is completely different between AMD and AD. Besides, even though Aβ is deposited both in the brain and in drusens, the phenotypic traits characterizing AD and AMD are quite dissimilar. Extracellular Aβ-containing deposits are observed as drusens into sub-RPE space in AMD and as senile plaques into the hippocampus and cortex (brain), ganglion cell layer (retina), and around retinal vessels in AD. The main cell types affected in AMD are the RPE cells, whereas in AD, the hippocampal neural cells are primarily damaged. Again, Aβ accumulation occurs outside the outer blood–retinal barrier in AMD and inside the blood–brain barrier (BBB) and blood–cerebrospinal fluid (CSF) barrier in AD. Collectively, these findings suggest that AMD should be better considered as a subtype of “amyloid disease” with several similarities and differences with AD (Ohno-Matsui, 2011; Wang and Mao, 2021).

## Neurotrophin Impairment in AD and AMD: The Neuroprotective and Regenerative Actions of NGF Delivery in the Brain and Eye

NGF is part, and indeed the pioneer, of neurotrophin family including brain-derived neurotrophic factor (BDNF), neurotrophin 3 (NT-3), and neurotrophin 4/5 (NT-4/5) (Huang and Reichardt, 2001; Calissano et al., 2010a, b; Cattaneo and Calissano, 2012; Aloe et al., 2015) expressed in mammalian brain and in the neural retina (Garcia et al., 2017). NGF is a pleiotropic factor that promotes the survival, growth, and differentiation of neuronal cells of the CNS and peripheral nervous system (PNS) during development and adulthood. Its “trophic” prosurvival activity has been more recently documented to consist of an anti-amyloidogenic action keeping under control the apoptotic program (Calissano et al., 2010a, b). NGF also exerts a modulatory role by acting on specific non-neuronal cells of the neuro-immuno-endocrine system (Aloe and Chaldakov, 2013). NGF and its cognate receptors (the high-affinity tyrosine kinase receptor TrkA and the low-affinity pan-receptor p75), in addition to their classical distribution in the cholinergic basal forebrain nuclei and in the cortical and hippocampal regions of the CNS, are also expressed in different ocular fluids and tissues, such as aqueous/vitreous humor, posterior lens, retina, and optic nerve (Micera et al., 2007). An imbalance between the NGF-dependent, TrkA-mediated survival, the growth actions, and the p75NTR-mediated activation of apoptosis/growth inhibitory pathway is causally associated with the onset and/or development of several neurodegenerative diseases of both CNS and PNS (Micera et al., 2004; Cuello et al., 2007, 2010; Garcia et al., 2017), including AD (Sofroniew et al., 2001; Schulte-Herbruggen et al., 2008; Cattaneo and Calissano, 2012) and AMD (Lambiase et al., 2009; Telegina et al., 2019; Esposito et al., 2021). In view of its strong neuroprotective and regenerative actions both *in vitro* and *in vivo*, delivery of NGF to the brain and eye has been always explored with therapeutic purposes for cerebral and extracerebral (retinal) neurodegenerative diseases, including AD and AMD (Gupta et al., 2016). Nevertheless, the clinical benefits of NGF therapy to the brain are restricted by its inability of bypassing the BBB to reach the CSF *via* the brain ventricles, poor pharmacokinetic profile, and nociceptive unwanted side effects due to the broad distribution of its receptors along the intrathecal space (Eriksdotter Jönhagen et al., 1998). All these pharmacological aspects of NGF drastically reduce its efficacy and safety when administered *in vivo*. In order to achieve a cerebral long-term delivery of NGF in biologically active therapeutic doses, multiple approaches have been employed to allow a sustained release/targeting of NGF both in the brain and eye. However, starting from the initial attempts of direct injection, the classical gene- and cell-based routes along with the more innovative nanotechnological strategies – including carrier-mediated release, polymer-based and encapsulated cell (EC) delivery system, nanoparticles, and quantum dots – have encountered several hurdles prior to becoming suitable treatments of cerebral and extracerebral symptoms of patients suffering from AD and, potentially, from AMD. The main therapeutic approaches for NGF delivery will be discussed in the following sections.

### Direct Intracerebral Infusion of NGF

To date, local and direct intracerebroventricular (ICV) injections of NGF in close proximity to the cholinergic neuronal soma located in the CNS basal forebrain have been carried out by implantation of fibroblasts or neuronal progenitors genetically modified to secrete NGF or by delivery of NGF-expressing adeno-associated virus both in animal models and in early clinical trials (Fischer et al., 1987; Olson et al., 1992; Seiger et al., 1993; Eriksdotter Jönhagen et al., 1998). Adult cholinergic neurons are successfully rescued from degeneration by NGF ICV delivery, and mnemonic recovery is greatly sustained *in vivo* following infusion of this neurotrophin as well (Tuszynski et al., 1990, 2005, 2015; Markowska et al., 1994, 1996; Martínez-Serrano et al., 1995; Castel-Barthe et al., 1996; Klein et al., 2000; Bishop et al., 2008). An intraparenchymal application of recombinant NGF protein into sites adjacent to degenerating cholinergic cell bodies of rodents is effective and well-tolerated at least up to a 2-week period of treatment (Tuszynski, 2000; Pizzo and Thal, 2004).

### Peripheral Administration of NGF Using Nasal and Intraocular Delivery

Even though the initial improvement of cholinergic functions following the direct intracerebral delivery of NGF *in vivo* turned out to be encouraging, the manifestations of an undesirable back pain and weight loss along with a low diffusivity of the drug within the brain have prompted researchers and companies to further discontinue this route of administration for therapeutic reasons (Faustino et al., 2017; Mitra et al., 2019). Thus, peripheral intranasal administration (INS) has provided an alternative approach to target *via* the olfactory path a sizeable amount of NGF directly to the brain, especially in a rodent model (Cattaneo et al., 2008). By taking advantage of the unique anatomic connections of the olfactory and trigeminal nerves linking the nasal mucosa and the CNS, INS delivery provides a high-vascularized absorption area favoring rapid penetration of NGF, but it does not overcome the occurrence of its adverse effects. Moreover, long-term INS of a drug causes damage to the nasal mucosa by altering the mucociliar activity (Malerba et al., 2011; Zhu et al., 2012). On the other hand, more recent studies have underscored the interesting possibility that the ocularly administered exogenous NGF (oNGF) is able to exert neuroprotective and/or regenerative properties on the retina and its brain projections, thus representing an innovative, safe, and non-invasive option for the cure of affected patients in the eye–brain system (Di Fausto et al., 2007; Lambiase et al., 2007; Tirassa et al., 2018). Furthermore, comparative studies carried out on adult and aged anti-NGF AD11 transgenic mice – an animal model characterized by AD-like neuropathology following chronic NGF antibody-mediated deprivation – have clearly demonstrated that ocular administration is less effective than intranasal delivery to protect cholinergic neurons and prevent behavioral deficits, even if used at higher doses (Capsoni et al., 2009; Covaceuszach et al., 2009). Likewise, intranasal delivery of NGF significantly improves the clinical outcome in children with neurological impairment following traumatic brain injury (TBI; Chiaretti et al., 2017).

### Gene- and Cell-Mediated NGF Delivery

Different approaches aimed at achieving a direct administration of exogenous NGF to the brain with beneficial outcomes have been recently provided by gene- and cell-based therapies (Faustino et al., 2017; Mitra et al., 2019). In this regard, preclinical and clinical studies have proved that the stereotactic surgical delivery of CERE-110 – an AAV serotype 2-based vector encoding for the human NGF – to the nucleus basalis of Meynert is a reliable and accurate neuroprotective strategy, being largely neurorestorative for a large part of cholinergic neurons localized in the rat fimbria-fornix lesion, in aged monkey models, and in human patients (Bishop et al., 2008; Mandel, 2010). Unfortunately, preliminary clinical studies designed to evaluate the actual safety and efficacy of the long-term administration of CERE-110 in subjects suffering from early-moderate AD (Rafii et al., 2014) are not followed by encouraging results. As reported in a recently published phase II clinical trial, no significant clinical rescue was detected by using AAV vectors expressing human NGF on 49 enrolled AD-diagnosed patients who underwent intracerebral injections of AAV-NGF or sham surgery, respectively (Rafii et al., 2018). In parallel with these findings, following 1-year lentivirus NGF gene delivery to the cholinergic basal forebrain, neither the systemic leakage of NGF and the occurrence of anti-NGF antibodies nor the activation of detrimental neuroinflammatory response in the brain with back pain or weight loss has been detected in aged non-human primates (Nagahara et al., 2009). Although the gene therapy procedures exploit recombinant AAV in which the viral genes for self-replication and incorporation of genetic material into chromosomal DNA have been deleted, the impossibility of modifying the dosage and/or interrupting the treatment once started along with potential toxicity and immunogenicity has raised concerns regarding the practicability, suitability, and usefulness of its application. To overcome the limitations of direct viral vector-mediated gene delivery, *ex vivo* gene therapy has been also evaluated. Neuronal stem cells (NSCs) transduced with human NGF by using a AAV2 vector, when grafted into the cerebral cortex of cognitively impaired rats undergoing chronic ICV infusion of the serine/threonine protein phosphatase (PP) inhibitor okadaic acid, are proven to successfully integrate into the host brain and to improve cognitive performance after transplantation (Wu et al., 2008). The NSC line overexpressing the human choline acetyltransferase (ChAT) gene recovers the learning/memory deficits and elevates the levels of acetylcholine (ACh) in CSF when transplanted into rat brain of an experimentally induced AD model, in which the application of ethylcholine mustard aziridinium ion (AF64A) specifically inactivates the cholinergic nerves. Interestingly, transplanted ChAT human NSCs migrate to different brain areas including the cerebral cortex, hippocampus, striatum, and septum of AF64A-cholinotoxin-lesioned AD rat model and successfully differentiate into viable and functional neurons and astrocytes as well (Park et al., 2012).

### Carrier-Mediated Sustained Release of NGF

Encapsulated cell biodelivery is an innovative strategy involving an *in vitro* genetically engineered human cell line which grows on a polymer scaffold behind a semipermeable filter and continuously releases a low but sufficient amount of therapeutic protein directly into a localized region of brain cells. This method combines the potency of gene therapy with the advantage offered by an implantable and retrievable device, acting as an actual biological micropump. In this regard, grafts of polymer-ECs modified to release NGF delay and/or prevent the degeneration of axotomized cholinergic neurons in the basal forebrain of rodents as well as of aged monkeys (Hoffman et al., 1993; Emerich et al., 1994; Kordower et al., 1994; Lindner et al., 1996). Implantation of encapsulated NsG0202, a clinical device which houses an NGF-secreting cell line (NGC-0295) derived from a human RPE cell line, is well-tolerated into the basal forebrain of Göttingen minipigs up to 12 months leading to an increase of NGF levels in the surrounding tissue (Fjord-Larsen et al., 2010; Wahlberg et al., 2012). EC-NGF biodelivery devices targeting the basal forebrain of implanted AD patients (*n* = 6) are safe in a 12-month study, leading to improvement in cognition assessed by clinical rating scales, EEG, MRI, and positron emission tomography (PET) (Eriksdotter-Jönhagen et al., 2012). Nevertheless, several drawbacks, such as the invasive nature of the procedures requiring hospitalization and neurosurgical intervention and their high costs, have discouraged the pursuing of both gene- and cell-based therapies as interventions to achieve a sustained and controlled release of NGF into the brain. An advancement in the field of NGF therapeutic strategies has been made with the contemporary development of intracerebral implants composed of biocompatible, synthetic, and natural polymers, including poly(ethylene co-vinyl acetate) (EVAc), poly(D,L-lactide-co-glycolic acid) (PLGA), polyanions (e.g., heparin, dextran sulfate, gelatin), and polycationic or polyanionic hydrogels which are endowed with different shapes and surface areas to provide high and localized doses of this neurotrophin into the brain (Hoffman et al., 1990; Powell et al., 1990; Johnson et al., 2008; Yang et al., 2009; Song B. et al., 2012; Hines and Kaplan, 2013). Nanoparticles have been more recently explored due to their multiple advantages, including small size (nanoscale dimensions), high solubility, multifunctionality, improved transport properties and pharmacokinetic profile, enhanced permeability, and retention. These parameters significantly enhance the penetrance of a drug into tissues through capillaries and also enhance its efficient delivery to target sites, thus providing a more powerful medicament for patients. Liposomes, polymeric micelles, dendrimers, magnetic nanoparticles, and quantum dots functionalized with NGF effectively deliver this neurotrophin both in neuronal cell culture and/or in animal model of AD leading to neurite outgrowth, diminution of cerebral Aβ accumulation, rescue in cholinergic functions, improved viability, and neuroprotection (Angelova et al., 2013; Zhao et al., 2016; Faustino et al., 2017; Marcus et al., 2018; Mitra et al., 2019; Tosi et al., 2020).

In summary, the pivotal role of NGF in the development, growth, maintenance, and plasticity of the CNS and PNS, both in normal and diseased conditions, represents a strong rationale and incentive of advancing innovative techniques to provide its localized and finely modulated delivery to target tissues.

## Ocular Administration of NGF as a Promising Therapeutic Approach to Counteract Degeneration of the Eye and Brain Both in AMD and AD Pathologies

The independent trophic and neuroprotective roles of NGF on the retina, optic nerve, and brain visual area and its direct and indirect projections to limbic structures, including the cholinergic hippocampus and septum, have been largely documented in different animal models and in paradigms of retinal injury and disease (Micera et al., 2004; Cattaneo and Calissano, 2012; Aloe et al., 2015; Garcia et al., 2017; Esposito et al., 2021).

In particular, under oxidative stress and inflammatory conditions similar to those early occurring both in AMD and AD, the activity of matrix metalloproteinase-7 protease is altered in the eye causing a local accumulation of proNGF and concomitant reduction of mature NGF followed by changes in the expression and function of its receptors (i.e., the survival TrkA receptor and the neurotrophin p75NTR receptor). Thus, even though it is still unclear whether ocular neuronal death is associated with the lack of neurotrophic support or impairment of its signaling events regardless of the level of the growth factor itself, ophthalmological clinical trials have taken advantage of the well-known anti-apoptotic action of NGF to slow and/or stop sight loss resulting from death of injured RGC or photoreceptors (Pardue and Allen, 2018; Hill et al., 2021). Compelling experimental evidence has documented the regenerative role of NGF on retinal ganglion cell survival in different animal models of optic nerve transection, ischemic injury, ocular hypertension, and glaucoma (Roberti et al., 2014). The impressive pharmacological potential of this neurotrophin in ophthalmic diseases has been recently brought to light by Guo et al. (2020) reporting that topical application of recombinant human NGF (rh-NGF) significantly reduces the RGC apoptosis *in vivo* following partial optic nerve transection (pONT) by protecting their soma and axons likely *via* the retrobulbar route (Guo et al., 2020). Intravitreal administration of rhNGF counteracts RGC degeneration occurring within 2 weeks following optic nerve crush (ONC) by reducing p75NTR and proNGF and by enhancing phosphorylation of TrkA and its intracellular signals at the retina level (Mesentier-Louro et al., 2019), in line with previous findings on another animal model of retinal disease, such as inherited retinitis pigmentosa (RP) (Sacchetti et al., 2017). The beneficial effect of NGF on injured eye has been also validated in RP experimental paradigm following retrobulbar injection (Lenzi et al., 2005) or in affected patients after eye drop administration (Falsini et al., 2016b). A randomized double-blind phase II clinical trial, aimed at testing the vision functions in 18 patients aged 2–23 years with stable disease and severe blindness caused by glioma, has proven that ocular instillation of NGF protects the optic pathway glioma-related visual impairment in the absence of any apparent side effects (Falsini et al., 2016a).

**FIGURE 2:**
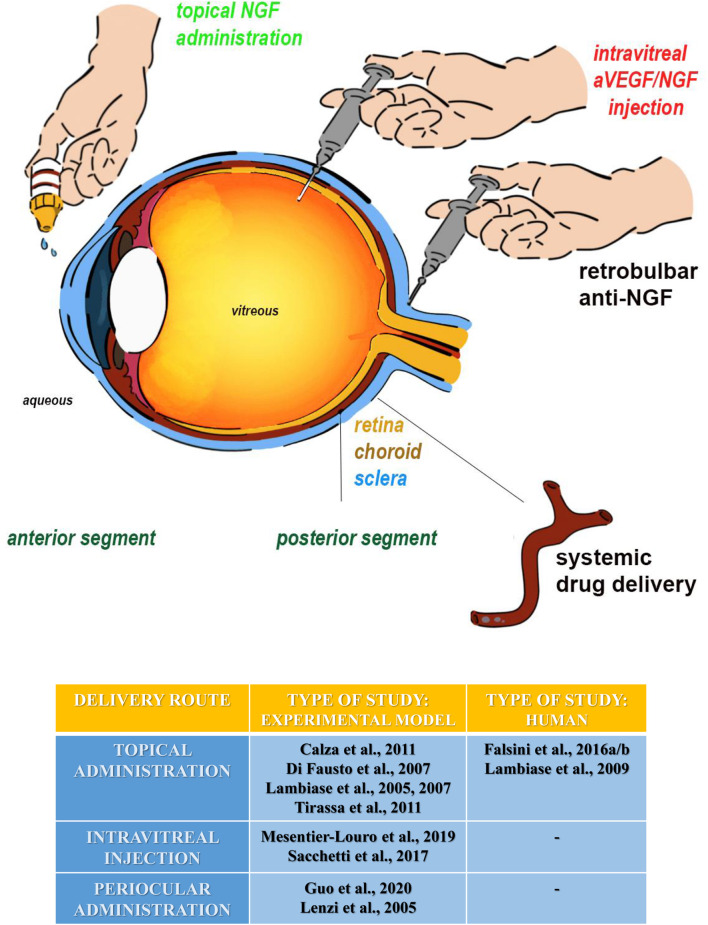
All ocular sites tested for experimental NGF delivery are summarized as follows: (1) the topical administration (eye drop instillation and emulsion/gel application) (green labeled), (2) the intravitreal injections (ophthalmic solutions/suspentions, implants, and nanotecnology), and (3) the periocular administration (implants or injections) at the posterior segment. The periocular route includes the subconjunctival, subtenon, peribulbar, and retrobulbar sites of injection. Except for the systemic route, all the others have been tested for NGF and anti-NGF at both experimental and clinical levels. The topical administration is actually a routine procedure for specific eye disorders. Highlighted in red: our future directions on experimental animals, comprising the anti-VEGF/NGF combined intravitreal injection or the use of NGF nanoparticles layered in the vitreal chamber. The table summarizes the different routes of NGF administration in the eye, in an experimental model and human beings.

More interestingly, a growing body of experimental and clinical studies have shown that administration of NGF in different ocular sites can be a feasible means to convey NGF and/or activate its downstream signals into the brain (Eftimiadi et al., 2021). Thus, the increase of the endogenous NGF availability, following its intraocular (intravitreal or periocular) exogenous injection and/or topic instillation (Figure 2), is currently exploited as a safe, non-invasive approach to successfully counteract retina and even brain neurodegeneration underlying clinical signs of AMD and AD phenotypes (Tirassa et al., 2018; Mitra et al., 2019). Consistently, when administered topically in animal models, NGF becomes available to the retina and optic nerve and exerts neuroprotective and/or regenerative properties on projections of the eye–brain system, including the nucleus basalis and septum as well (Lambiase et al., 2005, 2007). The local application of NGF drops using the ocular surface way induces an upregulation in the expression of NGF receptors and ChAT immunoreactivity (cholinergic markers) in chemically injured basal forebrain neurons of adult rats, indicating that eye NGF application somehow affects brain cells (Di Fausto et al., 2007; Lambiase et al., 2007). Along this line, the finding that a single ocular administration of NGF solution is sufficient to enhance the distribution of Ki67-positive cells (marker for proliferative cells) also expressing p75 neurotrophin receptors in the proliferating layer of the subventricular zone (SVZ) further supports the feasibility of ocular application of NGF in upregulating the development and maturation of neuronal progenitors in the CNS (Tirassa, 2011). Immunohistochemical studies have reported that an instillation of NGF as eye drops increases in a time-dependent manner the immunoreactivity of c-fos (markers for neuronal activation) in several areas of the limbic system and in primary visual centers of rats as well (Calza et al., 2011). Furthermore, an improvement in visual acuity and electrofunctional parameters has been found in a 94-year-old female affected with AMD 3 months after initiation of treatment with NGF and in the absence of any side effects up to 5 years of follow-up (Lambiase et al., 2009). Thus, despite intrinsic limitations due to proper physiology of the eye (tear turnover, nasolachrymal drainage, reflex blinking, ocular static, and dynamic barriers) which makes difficult a deeper drug penetration, the chance of exploiting in common clinical practice the ocular route to achieve a non-invasive distribution of several medicaments, including NGF, in therapeutic concentrations for the brain is gaining increasing interest to ocular scientists (Patel et al., 2013; Esposito et al., 2021). The advent of innovative nanotechnologies, new techniques, and devices (nanoparticles, nanosuspensions, liposomes, dendrimers, *in situ* gelling systems, intraocular implants, and microneedles) designed to overcome ocular barriers and side effects linked with conventional topical drops is currently allowing a more sustained drug release and an improved target specificity (Patel et al., 2013). Furthermore, the recent development of a genetically engineered NGF variant endowed with biological activity and reduced pain effect (painless NGF) further opens the expanding panorama of its applications *via* eye drop administration (Malerba et al., 2015; Testa et al., 2021).

Collectively, these recent and promising *in vivo* results underline that the protective and reparative actions of NGF used as eye drops are not only confined to the primary visual areas, but are also extended to other retinal central targets, including the forebrain structure (Calza et al., 2011; Tirassa, 2011). Moreover, these studies encourage investigations on the clinical effects of NGF therapy for the treatment of neurodegenerative diseases characterized by an imbalance in NGF/TrkA signal transduction pathway in the eye and in the brain, including AMD and AD, respectively (Mufson et al., 2008; Aloe et al., 2015; Canu et al., 2017; Esposito et al., 2021).

## Essential Trace Metals: Diagnostic Perspectives in AD and AMD

The common physiopathological aspects linking AD and AMD not only have important therapeutic implications but also can give additional insights into the development of novel biomarkers for both diseases. Besides, since retinal degenerative alterations manifest before the corresponding changes detected in the brain, quantification of ocular biomarkers will facilitate (i) the diagnosis of AD as well as AMD at their earliest stages when therapy is more likely to be effective in halting/slowing down symptomatology and (ii) a non-invasive monitoring of disease progression (Micera et al., 2019; Ong et al., 2019). In this context, a growing body of evidence has shown that dyshomeostasis of essential trace metals, namely, both depletion and excess of copper (Cu), iron (Fe), and zinc (Zn), causes severe damage to neurons and is causally associated with various neurodegenerative and retinal diseases, including AD and AMD (Bush, 2013; Faller et al., 2013; Micera et al., 2019).

In AD, a Cu imbalance is evident and associated with the misplacement of metal from cellular and tissue compartments: meta-analytic studies reported that Cu levels are decreased in the AD brain while increased in the periphery, suggesting a breakdown of Cu homeostasis control in the disease (Mathys and White, 2017). Elevated values of Cu in serum and decreased Cu values in the brain are associated with increased levels of Cu not bound to proteins and primarily to ceruloplasmin (the main Cu protein in serum) in general circulation: this Cu component, called non-ceruloplasmin Cu, also known as “free” Cu, is an established marker of Wilson disease, the paradigmatic disease of Cu toxicosis/accumulation. Non-ceruloplasmin Cu is an exchangeable, small molecular weight and filterable Cu component in the bloodstream that is toxic above a certain cutoff and can pass the BBB and distribute to brain parenchyma (Choi and Zheng, 2009; Squitti, 2012; Kepp and Squitti, 2019). Aβ40 and Aβ42 are flexible small α-helix and β-sheet structures representing the majority of Aβ forms in the AD brain. They can bind Cu(I/II) with relevant effects in initiating Aβ aggregation processes (Squitti, 2012; Mathys and White, 2017; Kepp and Squitti, 2019). Cu binding confers redox activity to Aβ (Squitti et al., 2017) that affects neuron viability (White et al., 1999). It has been recently hypothesized that a Cu dysfunction in the aging human brain might evolve as a gradual displacement of Cu from pools tightly bound to proteins to pools of loosely bound metal ions, involved in gain-of-function oxidative stress associated with Aβ peptides, a shift that might be provoked by chemical aging (Kepp and Squitti, 2019). The loss of Cu tightly bound to proteins may be associated with the loss of energy production and antioxidant function within neuronal cells, two important drivers of AD neurodegeneration (Kepp and Squitti, 2019). Non-ceruloplasmin Cu fits well in this construct since it is a loosely bound specie of Cu in general circulation and has been demonstrated to be a potential prognostic biomarker for conversion from mild cognitive impairment (MCI) to symptomatic AD, typified by copper imbalance (Squitti et al., 2014; Rozzini et al., 2018; Sensi et al., 2018). In AD, non-ceruloplasmin Cu reaches values similar to Wilson disease (Squitti et al., 2018) and has demonstrated a fair sensitivity in detecting which individuals with MCI will convert to Cu-AD (Squitti et al., 2014) and high specificity (95%) in detecting patients affected with Cu-AD due to copper exposure (Squitti et al., 2017), in line with preclinical models of the disease (Sparks and Schreurs, 2003; Singh et al., 2013; Yu et al., 2018; Hsu et al., 2019). Recently, several studies have shown that non-ceruloplasmin Cu might serve as a stratification biomarker for a subset of AD patients (Kepp and Squitti, 2019). The feature, called Cu-AD (Squitti et al., 2017), appears specific to a subset (50%–60%) of AD patients and is characterized by being a carrier of selective ATP7B gene variants (Squitti et al., 2013) and by having peculiar cortical activity and neuroimaging deficits (Kepp and Squitti, 2019).

Fe dysfunction has been strongly associated with AD pathogenesis in recent years. It is known that Fe imbalance and Aβ metabolism are synergistically regulated in neurodegeneration and can facilitate cellular processes which may result in AD development, pointing out to a central role of Fe dyshomeostasis in AD pathology. Recently, potential biomarkers associated with Fe imbalance have been individuated: ferritin, either in plasma or in CSF, has been shown to have potential in discriminating AD patients in their preclinical stage, categorized into low and high neocortical amyloid-β load, prior to cognitive impairment (Goozee et al., 2018). High ferritin levels suggest an increase of Fe abundance in the CSF and brain which can be associated with ferroptosis, an iron-dependent cell death that results from a buildup of lipid peroxides and is regulated by glutathione (GSH) peroxidase 4 (Acevedo et al., 2019). A recent study provided a very interesting theoretical significance of abnormalities of Fe associated with AD onset and progression (Ayton et al., 2021). Fe and Cu metabolism are intertwined in physiology, and they are certainly tied in AD (Squitti et al., 2010). Ceruloplasmin exemplifies the crosstalk protein between Cu and Fe balance because it is the main Cu protein in the body, and it controls Fe oxidative state: imbalance in one of the two metals has effects on the other (Siotto et al., 2016). Consistent with both Cu and Fe balance abnormalities, ceruloplasmin in the CSF has been shown to predict cognitive decline and brain atrophy in people with underlying Aβ pathology (Diouf et al., 2020). High levels of ceruloplasmin and the activation of the ceruloplasmin:transferrin (Cp:Tf) system, one of the main antioxidant systems acting in general circulation, were associated with worse cognitive performances and a severe medial temporal lobe atrophy, supporting the view that local iron accumulation in brain areas critical for AD might be strictly associated with Fe systemic alterations (Squitti et al., 2010). Recently, another point of conversion between Cu and Fe metabolism has been provided by the evidence of the loss in GSH levels in AD. GSH is one of the main endogenous free radical scavenger systems in the brain, acting as a co-substrate in GSH peroxidase-catalyzed reactions (Pinnen et al., 2011). GSH loss in the brain is linked to cognitive dysfunction (Currais and Maher, 2013) and is associated with Cu imbalance. This has been mainly stressed in preclinical models of AD induced by Cu exposure: high Cu and cholesterol levels trigger Aβ aggregation and plaque formation in the hippocampus and temporal cortex and impair learning and memory, thus recapitulating deficits featuring mental dementia (Yao et al., 2018).

Zinc homeostatic imbalance has been advocated as a driver of neurodegenerative processes, primarily linked to oxidative stress and the aging brain (Capasso et al., 2005; Frazzini et al., 2006). Somewhat reduced levels of zinc have been shown in AD through meta-analyses (da Silva et al., 2014; Ventriglia et al., 2015; Wang et al., 2015). The hypothetical role of this metal in AD pathology has been connected to neurotoxicity induced by mitochondrial production of ROS and by disruption of metabolic enzymatic activity (i.e., Cu, Zn superoxide dismutase activity) eventually leading to apoptosis and/or neurodegeneration (Li et al., 2017; Kabir et al., 2021). Zinc therapy, used in Wilson disease, effectively reduces non-ceruloplasmin Cu levels: this suggests potential beneficial effects in slowing the progression of cognitive decline in subsets of individuals who show both cognitive disturbances and signs of Cu imbalance (Squitti et al., 2020).

Concerning AMD, there is evidence that heavy metals might be involved in the pathogenesis of AMD (Ugarte et al., 2013; Micera et al., 2019). Beyond the deposition of Fe, Cu, and Zn metal ions in extracellular deposits of AMD, recent studies have shown accumulation of heavy intoxicant metals in eye tissues and in general circulation of AMD patients (Aberami et al., 2019). Major metal changes involve the accumulation of loosely bound Fe in human RPE, in Bruch’s membrane, and in photoreceptors located in the macula (Biesemeier et al., 2015; Dalvi et al., 2019). Fe plays a pivotal role in retina physiology as a cofactor of several processes of visual transduction: the expression of transferrin, ferritin, and ferroportin is upregulated in AMD macula (Baumann et al., 2017). A tight Fe control can avoid the accumulation of pro-oxidant and proinflammatory loosely bound species of the metal (Courtois et al., 2020). To contrast the accumulation of Fe in the aged retina of AMD patients, Fe chelation therapy has been proposed (Shu and Dunaief, 2018). Indeed, treatment with the Fe chelator deferiprone has been shown to be useful to prevent retinal degeneration (Song D. et al., 2012; Cui et al., 2020). However, several chelators (deferoxamine B) have been shown to cause adverse events (Micera et al., 2019). Recently, intravitreal injection of transferrin, a natural Fe chelator, has been demonstrated to have neuroprotective effects against oxidative stress in retinal degeneration: after injection in the vitreous, transferrin spans rapidly within the retina and accumulates in photoreceptors and in RPE, protecting retinal function from loosely bound Fe (Picard et al., 2015). Consistent with previous evidence (Micera et al., 2019), new findings show that Cu is increased in choroid-RPE of AMD. In contrast to Fe and Cu trends, Zn appears decreased in the aged retina and Zn supplementation has been proposed for AMD treatment (Gilbert et al., 2019). Zn supplementation has beneficial effects in reducing the progression of AMD according to the Age-Related Eye Disease Study (AREDS; Age-Related Eye Disease Study Research Group (AREDSRG), 2000; Chew et al., 2015; Seddon et al., 2016): daily supplementation with a formulation consisting of 500 mg vitamin C, 400 IU vitamin E, 25 mg zinc, 2 mg copper, 10 mg lutein, and 2 mg zeaxanthin (AREDS and AREDS2) was effective for slowing AMD progression (Kim et al., 2016). Cochrane reviews support the use of Zn for the delay of AMD progression and vision loss (Evans and Lawrenson, 2017; Micera et al., 2019). Beyond studies on essential trace metals, an increasing number of studies have been performed on circulating or local concentrations of intoxicant heavy metals, mainly linked to environmental or lifestyle risk factors. It is acknowledged that environmental and lifestyle factors affect the balance of bodily trace metals, and in particular, this occurs in the retina mainly through oxidative stress that originated from exposure of macular tissues to sunlight and local or systemic exposure to oxidative stressors, including smoke. In this line of research, important results have been gained mainly for cadmium (Cd) that, in most of these studies, has been found to be elevated in AMD patients, especially within the smoking population (Kim et al., 2016; Wu et al., 2014; Güngör et al., 2018). These findings are coherent with the results from histology studies, showing Cd accumulation in the retina and in the RPE, particularly in smokers (Wills et al., 2008). The proposed mechanisms of Cd uptake in the retina involve zinc transporters (ZIP4 and ZIP8) with high Cd affinity (Girijashanker et al., 2008). Studies on circulating intoxicant heavy metals have shown also increased levels of blood lead (Pb) (Güngör et al., 2018). Higher blood Pb and Cd levels in AMD (Güngör et al., 2018; Heesterbeek et al., 2020) together with increased levels of barium (Ba) have been found in neovascular AMD (236 patients with neovascular AMD compared with 236 age-matched controls). Conversely, chromium (Cr) concentration decreased. Interestingly, higher Cd concentrations have been found mostly in smokers. On this basis, preventive strategies targeting decreased Cd exposure have been postulated to reduce the burden of AMD, primarily directed to skip smoking (Heesterbeek et al., 2020).

## Conclusion

Alzheimer’s disease and AMD are brain and retinal degenerative diseases whose incidence is increasing worldwide due to lengthening of life expectancy. AMD and AD share several clinical and pathological aspects, including Aβ accumulation and aggregation, oxidative stress, inflammation, and alterations in local supportive/regulatory actions of NGF. Based on these similarities and in view of the strong evidence that NGF is endowed with regenerative actions on both the brain and the retina, a growing number of opportunities can be offered by the administration of this neurotrophin as a common therapeutic agent and neuroprotective strategy for AD and AMD. Currently, great efforts are being made to enhance the effectiveness of NGF-based therapy by exploring novel, safe, and reliable routes of its delivery to the brain and eye, ultimately assessed in preclinical and clinical trials. Alterations in the systemic and ocular levels of essential trace metals are currently evaluated as diagnostic and/or even predictive biomarkers for future precision medicine of both AD and AMD.

## Author Contributions

GA, RS, and AM designed and wrote the manuscript. VL, BB, MV, and PC contributed to the data, text, and edited the manuscript. All authors contributed to the article and approved the submitted version.

## Conflict of Interest

The authors declare that the research was conducted in the absence of any commercial or financial relationships that could be construed as a potential conflict of interest.

## Publisher’s Note

All claims expressed in this article are solely those of the authors and do not necessarily represent those of their affiliated organizations, or those of the publisher, the editors and the reviewers. Any product that may be evaluated in this article, or claim that may be made by its manufacturer, is not guaranteed or endorsed by the publisher.
